# Valorization of Spent Coffee Grounds Oil for the Production of Wax Esters: Enzymatic Synthesis and Application in Olive Oil Oleogels

**DOI:** 10.3390/gels10120817

**Published:** 2024-12-11

**Authors:** Aikaterini Papadaki, Vasiliki Kachrimanidou, Ioanna Mandala, Nikolaos Kopsahelis

**Affiliations:** 1 Department of Food Science and Technology, Ionian University, 28100 Argostoli, Greece; kpapadaki@ionio.gr (A.P.); vkachrimanidou@ionio.gr (V.K.); 2 Department of Food Science and Human Nutrition, Agricultural University of Athens, Iera Odos 75, 11855 Athens, Greece; imandala@aua.gr

**Keywords:** coffee wastes, circular economy, enzymatic catalysis, lipase, oleogelation, structured lipids, fat substitutes, olive oil

## Abstract

Spent coffee grounds, the main by-product of the coffee-brewing process, were valorized as a renewable source of lipids for the synthesis of novel wax esters and as an alternative and sustainable oil-structuring agent for the production of oleogels. The lipase-catalyzed reactions were implemented using fatty alcohols both under solvent-free conditions and with limonene as an environmentally friendly solvent. Wax esters were evaluated for their ability to formulate olive oil oleogels through the determination of the physical properties of oleogels. Results showed that high conversion yields were achieved when cetyl and behenyl alcohols were applied under solvent-free conditions, achieving a maximum yield of 90.3% and 91.7%, respectively. In the presence of limonene, the highest conversion yields were 88.9% and 94.5% upon the use of cetyl and behenyl alcohols, respectively. The behenyl wax esters exhibited greater oil-structuring properties, regardless of whether they were derived from solvent or solvent-free conditions. Rheological curves showed that the produced oleogels exhibited a strong gel strength, which was enhanced as the wax ester concentration increased. Frequency sweep curves confirmed the formation of a stable three-dimensional oleogel network and revealed the low dependence of the storage modulus on frequency. Overall, this study demonstrated that producing wax esters from renewable lipid sources has the potential to serve as an effective circular economy paradigm for creating novel oleogels with a broad range of applications.

## 1. Introduction

Oleogelation or oil-structuring describes the method employed to transform liquid oils to solid or semi-solid material, defined as oleogel. The resulting material has a gel-like character and appearance, but it is composed primarily of liquid oil (>90%) entrapped in a three-dimensional network [1]. In recent years, various factors, such as regulatory policies, increasing consumer demand for healthier food alternatives, and sustainability concerns, have intensified the search for alternatives to trans and saturated fats. Consequently, in response to regulations and consumer demands, the development of structured lipids has been extensively studied over the last decade, prompting the food industry to shift its focus towards oleogelation [2,3]. Specifically in the food industry, oleogels can be employed as fat replacers in several products, e.g., bakery, dairy, and meat products [4,5]. Oleogels can mimic the texture and the mouthfeel of fats while lowering the total fat content. As a result, healthier food products can be formulated, with reduced calories and improved nutritional profiles [6].

Two major groups of oleogels can be distinguished based on the oleogelator: low molecular weight oleogelators and high molecular weight oleogelators [7]. Noteworthy, the choice of the oleogelator can determine the mechanical properties, stability, and performance of the resulting oleogel. Specifically in food manufacturing, natural waxes (e.g., carnauba and candelilla), ethyl and methyl cellulose, alcohols, or esters of fatty acids constitute the leading oleogelators [8,9]. Besides natural waxes, wax esters can be also synthesized by lipases through transesterification reactions. The enzymatic process offers the advantage of sustainably producing wax esters by utilizing renewable oil sources. In fact, food waste and by-product streams could serve as alternative renewable oil resources for the enzymatic production of wax esters and other oleochemicals [10]. In addition to that, various food wastes and by-products could be used as the feedstock of the fermentation process towards the production of high yields of microbial lipids [11,12,13,14], which can be further converted into wax esters [15]. Enzymatic catalysis holds significant promise for generating natural-claimed products and reducing environmental impact. In enzymatic reactions, water is typically the main solvent; however, it presents disadvantages such as solubility challenges, the need for complex product purification processes, and difficulties in enzyme recovery. In this context, the use of organic solvents offers a potential solution to these limitations, making enzymatic methods more viable for industrial applications [16]. For instance, limonene, a bio-derived solvent primarily obtained from citrus peel waste, presents a promising alternative medium for enzymatic processes, offering the advantage of maintaining both safety and environmental compatibility. Notably, limonene has already been highlighted as an effective “green” solvent that enhances the enzymatic synthesis rate of hexyl laurate by Novozyme 435 lipase compared to conventional solvents [17]. Ultimately, this approach will endeavor to shift consumer perception towards products with reduced environmental impact that foster food system resilience [18,19].

Spent coffee grounds (SCGs) refer to the solid residues inevitably obtained as a by-product stream of coffee brewing or the production of instant coffee. The disposal or incineration of SCGs in landfills has a considerable environmental impact, as its high content of caffeine, tannins, and polyphenols forms a toxic residue [20]. Currently, the estimated global SCGs production of 60 million tons per year highlights the importance of developing effective strategies to valorize SCGs within the framework of the circular economy [21]. Previous studies have elaborated on the ultimate and proximate composition of SCGs, indicating the diverse portfolio of valuable compounds that could be exploited within the concept of biorefining [20,22]. These products include lipids, proteins, polyphenols, lignin, cellulose, and hemicelluloses, which could be further valorized toward the production of a wide spectrum of added-value products, such as animal feed, fuels or pellets, compost, enzymes, or even carotenoids and polyhydroxyalkanoates through biotechnological processes [20,23]. The oil content of SCGs is approximately 15% (*w*/*w*), and, therefore, the lipid fraction represents a valuable renewable resource. Currently, lipids of SCGs are utilized in transesterification reactions for biodiesel production, as rejuvenators for asphalt binders, and in cosmetic formulations [20]. Although there are various approaches for the valorization of SCGs, research must also prioritize the evaluation of the sustainability impact of these processes. From an environmental point of view, Forcina et al. [21] underlined that the main constraint for the reuse of SCGs is the absence of an effective collection system. Additionally, techno-economic studies have highlighted that achieving economically attractive and environmentally sustainable biodiesel production from SCGs requires centralized, large-scale production combined with the simultaneous extraction of high-value products [24,25]. Hence, alternative applications of SCGs oil should also be explored to enhance the sustainability of SCGs biorefining by diversified end-applications. 

In this context, the aim of this study was to evaluate the oil fraction extracted from SCGs as a raw material for the enzymatic synthesis of wax esters and their subsequent utilization to formulate olive oil oleogels. In our previous study, SCGs oil was incorporated in whey protein edible films to enhance their hydrophobic and antioxidant properties, suggesting one of its possible applications in the food industry [26]. Hence, this study aims at the valorization of SCGs oil towards the synthesis of wax esters and their further application for the development of novel olive oil oleogels. Under this concept, it can be expected that the economic feasibility of biorefining SCGs could be enhanced via the formulation of a multitude of end-products that will foster the pillars of a bio-based economy. Overall, this study is the first to report the production of wax esters from SCGs oil and their potential application as oleogelators for the development of novel structured lipids.

## 2. Results and Discussion

### 2.1. Enzymatic Synthesis of Wax Esters Using SCGs Oil

**Figure 1:**
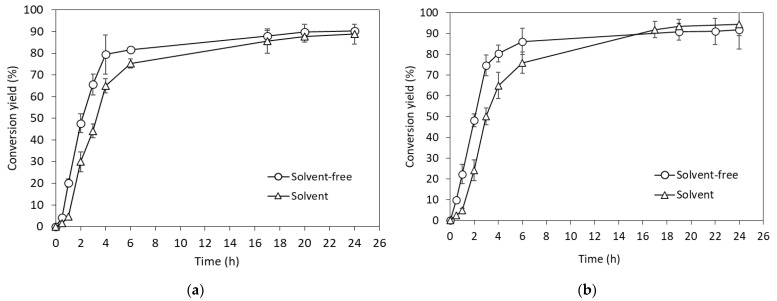
Wax esters enzymatic synthesis using spent coffee grounds oil with (**a**) cetyl alcohol and (**b**) behenyl alcohol, under solvent and solvent-free conditions.

SCGs oil was valorized towards the enzymatic production of wax esters. More specifically, transesterification reactions of SCGs oil were conducted with behenyl and cetyl alcohols, catalyzed by the commercial lipase (Novozyme 435). The results in Figure 1 illustrate the conversion yield of SCGs oil to cetyl and behenyl wax esters that reached a maximum of 90.3% and 91.7%, respectively, in solvent-free conditions. Moreover, the addition of limonene, as a “green” solvent, was assessed regarding the effect on enzymatic catalysis. In fact, limonene supplementation resulted in comparable conversion yields but allowed the enzymatic reactions to be conducted at lower temperatures due to improved substrate solubility. Hence, as can be observed in Figure 1, the highest conversion yields of oil to cetyl wax esters and behenyl wax esters were 88.9% and 94.5%, respectively. Notably, the conversion yields of all enzymatic reactions immensely increased during the first 6 h of the reaction and then were gradually stabilized at their maximum values. Zero-order kinetics for these first reaction hours showed that the reaction rate was higher in solvent-free conditions than in the presence of limonene (Table 1). Generally, hydrophobic solvents, showing a partition coefficient of log*P* > 4, increase the solubility of alcohols and triglycerides [27]. In this case, limonene was used as a “green” hydrophobic solvent (log*P* = 4.5), which increased substrate solubility, allowing for the use of lower reaction temperatures. However, the kinetics showed that the substrate conversion yield was reduced in the presence of limonene, probably due to substrate dilution. In this case, limonene might have caused the lower reaction rates due to the decreased collision frequency between the substrate and the enzyme [27,28].

### 2.2. Production of Oleogels Using Wax Esters Synthesized from SCGs

**Figure 2:**
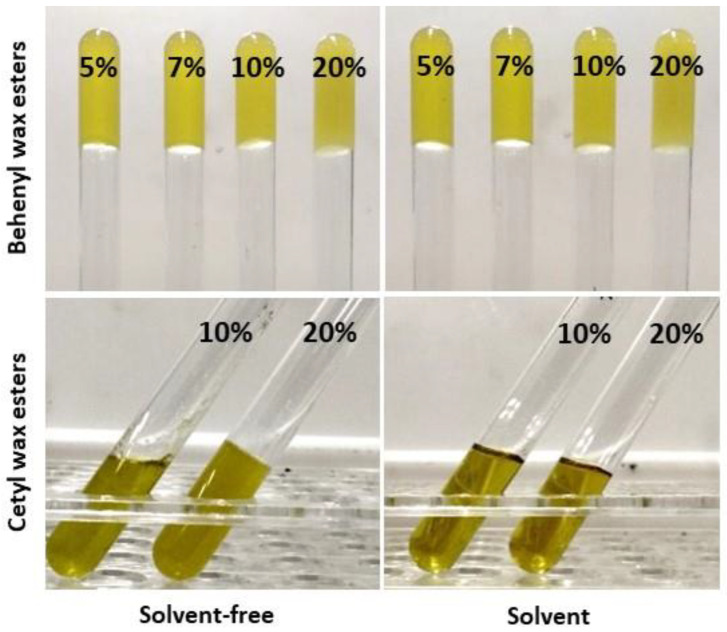
Visualization of oil-gelling effect using varying concentrations of behenyl wax esters and cetyl wax esters produced by spent coffee grounds oil under solvent-free and solvent (limonene) conditions.

In the forthcoming step, all the wax esters obtained from SCGs were evaluated as olive oil-structuring agents. In particular, cetyl and behenyl wax esters derived from both solvent and solvent-free reaction conditions were used in varying concentrations (5, 7, 10, and 20% *w*/*w*), and the formation of oleogels was tested by turning over or inclining the mixtures. As illustrated in Figure 2, behenyl wax esters demonstrated better gelling capacity than cetyl wax esters, leading to the macroscopic formation of oleogels at all concentrations tested. Conversely, when cetyl wax esters were applied, a minimum quantity of 20% (*w*/*w*) was required to form a stable three-dimensional structure, and this was achieved only under solvent-free conditions. Similar results have been obtained in previous studies dealing with oleogel formation using enzymatically produced wax esters from soybean fatty acid distillate [10]. Based on these results—and considering that a high amount of waxes may lead to undesirable sensory attributes in the food product (e.g., a waxy mouthfeel) [29,30] and that low concentrations (below 10%) of the oleogelator are typically preferred for oleogel production [1,31]—the subsequent steps of this study focused on characterizing oleogels structured with behenyl wax esters at concentrations of 5%, 7%, and 10% (*w*/*w*). Specifically, from this point forward, oleogels structured with behenyl wax esters derived from solvent-free conditions and limonene-solvent conditions are denoted as B5, B7, and B10, and BL5, BL7, and BL10, respectively.

### 2.3. Oil Bind Capacity (OBC) and Color Parameters of Oleogels

**Figure 3:**
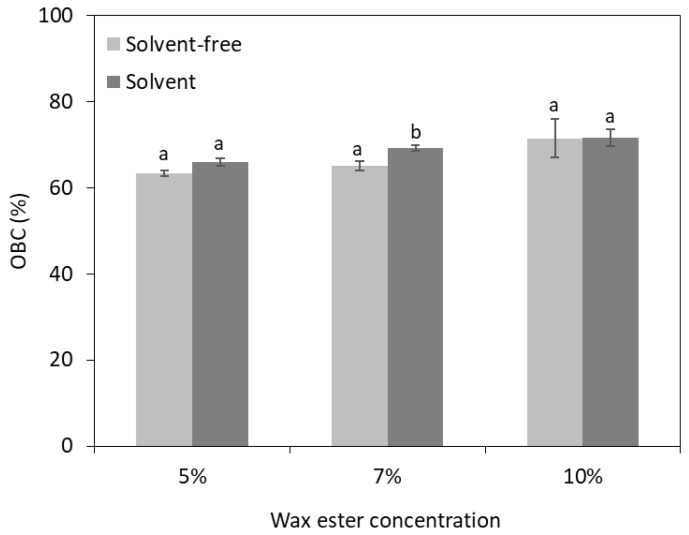
Oil binding capacity (OBC) of olive oil oleogels structured with varying concentrations of behenyl wax esters produced by spent coffee grounds oil under solvent-free and limonene-solvent conditions. Different letters on the bars indicate significant differences between oleogels with the same wax ester concentration (*p* < 0.05).

The OBC is a substantial parameter for assessing the stability of the oleogel. For instance, the formed oleogels should withstand processing conditions (e.g., mixing and shear stress) and retain the oil in the crystalline structure [32]. In this study, the OBC of the oleogels increased gradually as the concentration of SCGs behenyl wax esters increased, whereas the effect of limonene-derived wax esters was negligible (Figure 3). The minimum OBC was 63.2%, whereas the highest one was 71.5%, at 5% and 10% wax ester concentrations, respectively. The increasing OBC values observed with higher oleogelator concentrations have also been reported for olive oil oleogels structured with carnauba wax and monoglycerides [33]. The use of natural waxes seems generally to provide higher OBC values to oleogels compared to enzymatically produced wax esters. For example, olive oil oleogels formulated with beeswax and sunflower wax demonstrated over 99% OBC, regardless of the oleogelator concentration [34]. Similarly, the addition of 3% and 9% candelilla wax to formulate olive oil oleogels resulted in OBC values ranging from 61.7% to 91.8%, respectively [35]. Nevertheless, the lower OBC values obtained in the present study are comparable to those of commercial margarines, which exhibit OBC values between 70% and 79% [36]. Moreover, taking into account the possibility of upgrading and tailoring the properties of the oleogels with binary mixtures, these results provide the foundation for forthcoming research, given the sustainable character of the process. For instance, a mix of oleogelators could improve the OBC by altering the intramolecular forces and the crystalline structure [37].

Color analysis revealed that all oleogels had comparable color parameters, as illustrated in Table 2. Statistical analysis showed no significant differences (*p* > 0.05) between the different oleogels for each color parameter. Similar findings were observed in a previous study for extra virgin olive oil and carnauba wax oleogels, with L*, α*, and b* ranging from 42 to 54, −5.6 to −6.4, and 16 to 25, respectively [34]. Consumer acceptance is essential and can only be achieved if the substitute provides a comparable sensory experience or offers other significant benefits [38]. In line with this, the color parameters are also crucial for successful product acceptance. The produced oleogels have similar color parameters with other wax-based oleogels [10] but also with commercially available structured lipids, such as margarine [33].

### 2.4. Rheological Behavior of Oleogels: Amplitude Sweep Tests

**Figure 4:**
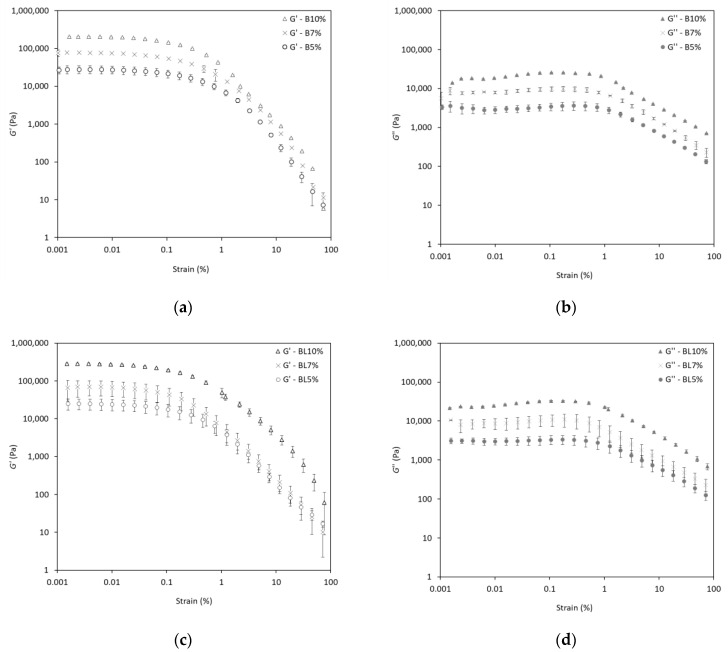
Storage modulus (G′) and loss modulus (G″) during amplitude sweep tests of olive oil oleogels structured with varying concentrations of (**a**,**b**) behenyl wax esters produced under solvent-free conditions (B) and (**c**,**d**) behenyl wax esters produced under limonene-solvent conditions (BL).

The viscoelastic properties of the oleogels were analyzed using small deformation oscillatory tests (amplitude and frequency sweep tests). In this section, the changes in G′ and G″ during the strain sweep experiments are discussed. The results presented in Figure 4a–d show two distinct regions: a linear and a non-linear. In the linear viscoelastic region (LVR), all oleogels demonstrated higher G′ than G″ values, indicating a clear predominance of the elastic behavior, hence the existence of a firm gel-like network structure. Moreover, an increase in the concentration of wax esters from 5% to 10% entailed an increase in G′, suggesting a stronger crystalline structure. In particular, G′_max_ increased nearly 10-fold when 10% wax esters were applied to the oleogels, compared to 5% wax esters. As a matter of fact, it has been reported that the use of higher amounts of wax esters results in the formation of a strong gel, also expressed with high G′ values [39,40]. Regarding the non-linear region, decreasing G′ and G″ values are observed until the crossover point (G′ = G″). At the crossover point, also known as the flow point, a disruption of the internal structure occurs, and the oleogel presents a liquid-like behavior [41]. The limiting G′ values of LVR (G′_LVR_) are presented in Table 3. Notably, as the wax concentration increased, the G′_LVR_ values also increased, thus confirming the formation of a stronger oleogel structure at increased wax ester concentrations. However, a similar critical strain was determined for all oleogels, indicating their identical LVR, which demonstrates their similar resistance to structural deformation regardless of the wax ester concentration. As previously reported, an ideal G′ for soft to hard solid fats should fall within the range of 10^2^ to 5 × 10^3^ kPa [42]. In our case, oleogels with 10% SCGs wax esters presented soft fat behavior (Table 3).

### 2.5. Rheological Behavior of Oleogels: Frequency Sweep Tests

**Figure 5:**
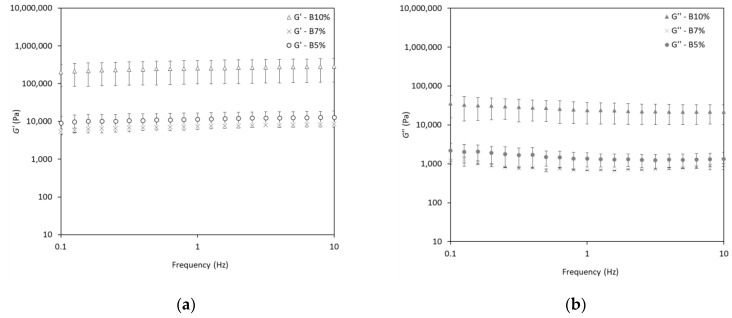

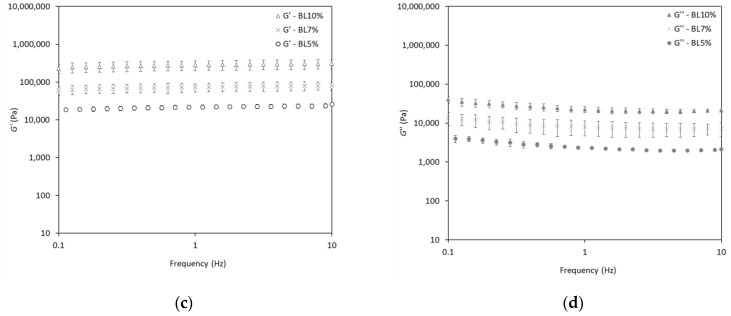
Storage modulus (G′) and loss modulus (G″) during frequency sweep tests of olive oil oleogels structured with varying concentrations of (**a**,**b**) behenyl wax esters produced under solvent-free conditions (B) and (**c**,**d**) behenyl wax esters produced under limonene-solvent conditions (BL).

The solid-like behavior (G′ > G″) of the oleogels was also evident in the frequency sweep tests (Figure 5a–d), which were conducted within the LVR. The G′ and G′′ curves were linear and none of them presented the crossover point. In particular, the behavior of modulus with increasing frequency is useful for the classification of oleogels into either strong or weak gels. Strong gels exhibit a frequency-independent pattern, while weak gels demonstrate a frequency-dependent pattern [31]. As illustrated in Figure 5a–d, all oleogels exhibited frequency-independent characteristics, with the G′ remaining constant as the frequency increased. These observations are typical of strong gels with good tolerance to the rate of deformation [43,44]. Interestingly, oleogels containing 10% behenyl wax ester concentrations presented higher viscoelastic moduli than the other two concentrations (5 and 7%) in the tested frequency range. These findings demonstrated the strong relationship between the oleogelator concentration and the viscoelastic properties. In particular, these results are consistent with a previous study that reported gel strength improvement with an increase in its solid volume fraction [1]. Furthermore, the independence of the viscoelastic properties from the applied frequency was also demonstrated by the *Power Law* parameters obtained after curve fitting (Table 3). Notably, the use of a higher wax concentration resulted in higher *n* values of the oleogels. The highest *n* values were observed for oleogels containing 10% wax esters, regardless of whether they were produced under solvent or solvent-free conditions. This is explained by the fact that the constant coefficient (*n*) is the extent of G′, thus the higher values of *n* are proportional to the oleogel strength [45]. Additionally, the flow behavior index (*m*) reveals the frequency dependency of G′. In the present study, all *m* values were close to zero values, which indicates the highly elastic behavior of the oleogels [41]. Consequently, the produced oleogels showed low dependency to the frequency, which is a typical behavior of a strong gel network. 

Although higher wax concentrations produced firmer oleogels, the loss tangent value at 1 Hz was similar across all samples (*p* > 0.05), ranging from 0.08 to 0.12. The low loss tangent values of the oleogels are associated with their gel-like structure and strength, whereas higher values, closer to 1, indicate a weaker gel strength [46]. Loss tangent values below 1 determine the predominance of the elastic character and values above 1 determine the viscous or liquid state of the material. Consequently, the produced oleogels exhibited a stable three-dimensional network structure, as indicated by their sufficiently low loss tangent values.

### 2.6. Rheological Behavior of Oleogels: Temperature Ramp Tests

**Figure 6:**
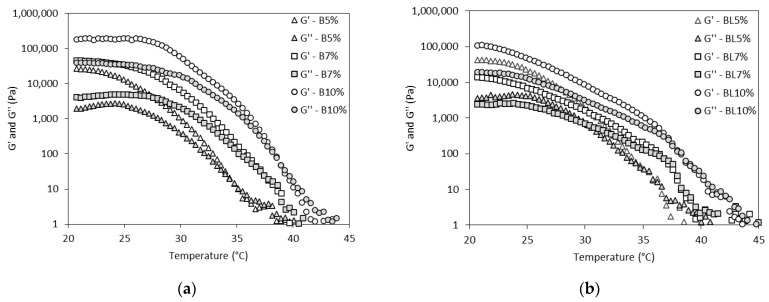
Storage modulus (G′) and loss modulus (G″) during temperature sweep tests of olive oil oleogels structured with varying concentrations of (**a**) behenyl wax esters produced under solvent-free conditions (B) and (**b**) behenyl wax esters produced under limonene-solvent conditions (BL).

**Figure 7:**
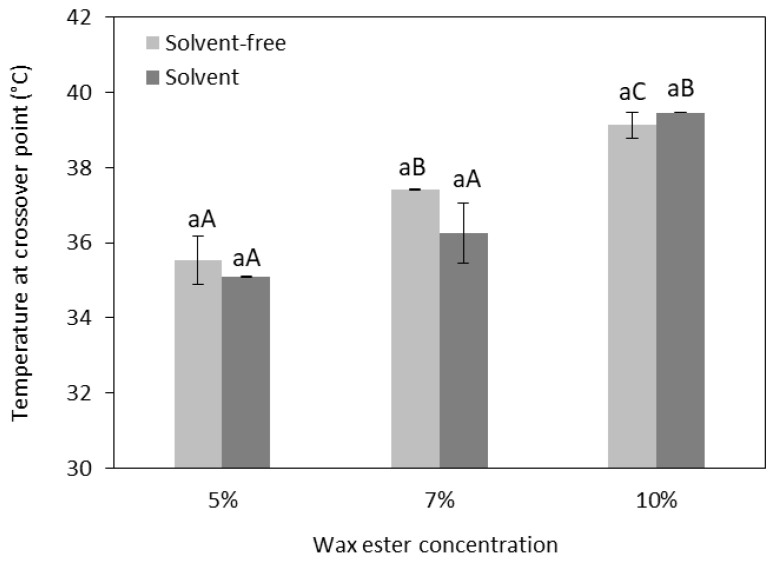
Temperature at the crossover points of olive oil oleogels structured with varying concentrations of behenyl wax esters under solvent-free and solvent (limonene) conditions. Different letters on the bars indicate significant differences between oleogels (*p* < 0.05). Lowercase letters refer to oleogels with the same wax ester concentration and at different solvent conditions. Uppercase letters refer to oleogels with different wax ester concentration at each solvent conditions.

The viscoelasticity of the oleogels was also studied during temperature ramp tests, and the results are illustrated in Figure 6. The impact of heat on the oleogels was assessed by gradually increasing the temperature from 20 °C to 80 °C. In the temperature range from 20 °C to 35–38 °C, the solid-like behavior of oleogels predominates (G′ > G″). Beyond this temperature range, the oleogels lose their characteristic solid-like structure. The crossover points, where the oleogels acquire deformation, were observed at 35 °C for oleogels with 5% wax esters (Figure 7). As the concentration of wax esters increased, the temperature at the crossover point also increased, showing a maximum of 39 °C for oleogels with 10% wax esters. A further rise in temperature led to permanent structural changes as G′ was lower than G″, and under these conditions, oleogels behave more like a liquid material. Additionally, Figure 7 shows that there were no statistical differences between the crossover points of oleogels (*p* > 0.05) structured with wax esters from solvent or solvent-free conditions.

As has been demonstrated, the rheological behavior of wax-based oleogels is strongly affected by the crystal morphology. In fact, crystal morphology is highly dependent on the type of waxes and, more specifically, on their chemical composition, polarity, chain length, and melting point. A recent study revealed a strong dependency of the G*_max_ on the crystal size of wax esters with high chain length, concluding that crystal size defines the strength of the oleogel [47]. The high chain length of behenyl wax esters may have positively affected the crystal morphology, resulting in oleogels with high G′ values. Moreover, a previous study showed that oleogels prepared with high concentrations of natural waxes, such as rice bran wax, demonstrated high G′ values due to the greater crystal entanglement [48]. Consistent with this, it was observed that the G′ of olive oil oleogels at the crossover points (Figure 7) gradually increased with increasing wax ester concentrations.

Generally, the behavior of the oleogels across an increasing temperature range provides valuable insights into their spreadability. In fact, spreadable margarine products must retain their gel-like structure even at temperatures above the refrigerated storage conditions [36]. In this study, the crossover points of olive oleogels were determined at high temperatures, suggesting that they can maintain their solid structure even outside refrigerated storage conditions. Moreover, the crossover point reflects changes in the crystal network of the oleogels and serves as an indicator of their melting temperature [49]. Additionally, the melting behavior at temperatures around the human temperature demonstrates oleogels with desirable melting profiles for food applications. In fact, melting properties are crucial in the production of fat-based food products, with the main objective being to achieve a melting point that is near human body temperature, as this is a key factor for consumer satisfaction.

### 2.7. Texture Analysis of Oleogels

Table 4 shows the results of texture analysis of the oleogels during storage at 4 °C. As one can observe, firmness was significantly increased (*p* < 0.05) as the wax concentration increased. The lowest firmness was 0.35 N and raised up to 0.95 N when the oleogels contained 10% behenyl wax esters. It is also worth noting that oleogels prepared with wax esters derived from either solvent-free or solvent conditions, showed no significant differences in firmness values (*p* > 0.05). The firmer texture of oleogels with 10% wax esters can be also correlated with the higher G′ of these oleogels, as indicated in the rheological analyses (Section 2.4 and Section 2.5). Commercial fat products, such as margarine, have an average firmness of up to 1.6 N [50], which is relatively close to the firmness obtained for olive oil oleogels with 10% wax esters.

Previous studies have shown that both softer and firmer gels can result from the use of natural and enzymatically produced wax esters. These variations seem to be influenced by the origin of both oils and oleogelators, as well as the chemical interaction between them [49]. It has also been reported that oil polarity critically affects the strength of the wax-based oleogels [29]. Öǧütcü and Yilmaz [33] reported a firmness of about 5.4 N (stored at 4 °C) for the olive oil oleogel structured with 10% carnauba wax, whereas in another study, soybean oil oleogel structured with 5% candelilla wax had a firmness of 0.9 N [51]. Concerning other enzymatically produced wax esters, previous studies showed that soybean fatty acid distillate (SFAD) or microbial oil (MO) derived wax esters resulted in olive oil oleogels with higher firmness (about 2–2.5 N after 20 days stored at 4 °C). In addition to that, different chemical compositions of oils can significantly influence the firmness of oleogels. For instance, in our previous study, MO-derived wax esters formulated a soft soybean oil oleogel (1.9 N), whereas the same oleogelator produced a firmer microbial oil oleogel (14.5 N) [49]. This suggests that different oil oleogelator mixtures contribute to distinct textural properties. Furthermore, the degree of unsaturation and the fatty acid composition of the oil play an important role in the chemical interaction between wax and oil. In view of the above, the firmness of olive oil oleogels structured with wax esters obtained from SCGs appears to be mainly influenced by the high unsaturation degree of the extra virgin olive oil [52].

## 3. Conclusions

In this study, SCGs oil was valorized towards the enzymatic synthesis of wax esters using two fatty alcohols, namely behenyl and cetyl alcohol, under solvent-free and “green” solvent conditions. The highest wax ester conversion yield, reaching 94.5%, was obtained during enzymatic synthesis in the presence of limonene as the “green” solvent. Moreover, the reaction kinetics proved that limonene contributed to higher substrate solubility and accelerated enzymatic reactions, confirming its suitability for enzymatic fatty acid bioconversions. Subsequently, the oil-structuring properties of the produced wax esters were determined through the preparation of olive oil oleogels. Oleogelation was successfully achieved by the addition of SCGs behenyl wax esters, even at low concentrations. The olive oil oleogels formulated with behenyl wax esters derived from either solvent-free or limonene-solvent conditions presented similar OBC, rheological behavior, and firmness. In fact, oleogels with 10% SCGs wax esters demonstrated the strongest gel properties network; however, even the lower concentrations (5 and 7%) contributed to olive oil oleogels with firm gel-like structures. Furthermore, temperature ramp tests demonstrated that oleogels deformation occurred within the temperature range of 35–39 °C, indicating their desirable melting profile for the formulation of fat-based food products. More specifically, the properties of the olive oil oleogels revealed their potential application in spreadable soft fat-based products, such as butter alternatives. 

As oleogels hold great potential as healthy fat alternatives, scaling up their production to an industrial level is essential to deliver their benefits to society. However, their industrial commercialization and the environmental impact of the oleogelation process have not yet been thoroughly addressed. The commercialization of oleogel production must consider several factors, including the oleogelation protocol. In this context, the overall scalability and sustainability of oleogels have recently been evaluated based on several factors, including heat treatment, electrical energy consumption, and oleogelation time. More specifically, oleogels were categorized as low-, medium- and high-input, with the latter requiring the highest amount of at least one input, which makes them challenging to scale up [53]. According to this classification, the hot direct oleogelation method utilized in this study, which employed wax esters from SCGs oil and operated at a cooling rate ≥ 3 °C/min, can be categorized as a low-input approach.

In conclusion, this study demonstrated that the produced oleogels exhibited promising properties for food applications. Moreover, from both industrial and sustainability perspectives, the oleogels demonstrated significant potential for scalability.

## 4. Materials and Methods

### 4.1. Materials and Reagents

SCGs from the Arabica variety were obtained from a local coffee shop (Argostoli, Kefalonia, Greece) and stored at −20 °C until further use. Extra virgin olive oil was purchased from a local food store (Argostoli, Kefalonia, Greece). Behenyl and cetyl alcohols (>99% and ≥95% purity, respectively), as well as the commercial lipase Novozyme 435 (≥5000 U/g, lipase B from Candida antarctica immobilized on acrylic resin), were purchased from Sigma-Aldrich (Sigma-Aldrich, St. Louis, MO, USA). All other reagents were of analytical grade.

### 4.2. Enzymatic Synthesis of Wax Esters

SCGs were initially oven-dried at 40 °C until moisture content was 7%, and oil extraction was conducted using hexane at a solid–liquid ratio of 1:9 at 25 °C for 24 h (twice) [26]. Vacuum evaporation was employed to recover SCGs oil, which was utilized for wax ester production via an enzymatic route, as described in our previous studies [15]. Briefly, SCGs oil with cetyl alcohol or behenyl alcohol was utilized at a molar ratio of 1:3 (oil–alcohol). As the melting points of fatty alcohols are different, the reactions were performed at 50 °C for cetyl alcohol and at 70 °C for behenyl alcohol in a water bath. Enzymatic synthesis was performed in a solvent-free system under agitation, and the reaction was initiated by the addition of 10% (*w*/*w*, based on oil amount) of lipase. Enzymatic reactions were also conducted under “green” solvent conditions using limonene (30%, *w*/*w*) [54]. In this case, the reactions were conducted at lower temperatures, i.e., 40 °C for cetyl alcohol and 60 °C for behenyl alcohol, due to easier solubilization of alcohols in the presence of limonene [54]. Samples were withdrawn at specific time intervals to assess the wax ester production. The quantification of wax esters was carried out using gas chromatography, following a previously described protocol [15]. Additionally, the zero-order model was applied to describe the kinetic behavior of enzymatic synthesis and to determine the reaction rate constant (%/h) during the first reaction hours.

### 4.3. Oleogels Production

Oil-gelling properties of the wax esters were investigated through the preparation of olive oil oleogels. In particular, olive oil oleogels were prepared using different concentrations (5%, 7%, 10%, and 20%, *w*/*w*) of cetyl and behenyl wax esters, which were derived from enzymatic synthesis. Olive oil and wax esters were precisely weighed and the mixture was kept at 90 °C under agitation for 10 min until all the components melted [48]. Oleogels were transferred into screw-capped glass vials and placed in ice to cool until the temperature of the oleogel reached 10 °C (average cooling rate of 4.9 °C/min), allowing gel formation. The oleogels were designated based on their oleogelator and the solvent conditions used during wax ester synthesis. Likewise, oleogels structured with 5%, 7%, and 10% behenyl wax esters, synthesized under solvent-free conditions, were labeled as B5, B7, and B10, respectively. Similarly, those prepared under limonene conditions were denoted as BL5, BL7, and BL10.

### 4.4. Characterization of Oleogels

#### 4.4.1. Color

Oleogels were poured in cylindrical tubes (2 × 1 cm, diameter × height) and their color was determined using a colorimeter (ChromaMeter CR-400/410, Konica Minolta, Japan). The colorimeter was initially calibrated using a white and a black plate. Subsequently, the color of the oleogels was measured using the CIE-L*a*b* uniform color space (CIE-Lab), where L* represents lightness, a* denotes the green (−) to red (+) axis, and b* denotes the blue (−) to yellow (+) axis. Additionally, the parameters hue angle (h°) and Chroma (C*) were recorded [10,55].

#### 4.4.2. Oil-Binding Capacity (OBC)

The OBC of oleogels was conducted according to the protocol described by Choi et al. [56], with slight modifications. Briefly, samples were precisely weighed (2 g) into conical tubes, centrifuged (15 min, 10,000 rpm), and then inverted onto filter paper to drain for 24 h. Then, the OBC of oleogels was calculated according to the equation:

(1) $$OBC\;(\%)=100-\left(\frac{w_{1\;}-w_{2\;}}{w_{1}}\times 100\right)$$

where *w*_1_ represents the weight (g) of oleogel before centrifugation and *w*_2_ represents the weight (g) of oleogel remaining after centrifugation and drainage.

#### 4.4.3. Rheological Properties

The Discovery HR3 hybrid Rheometer (TA Instruments, New Castle, DE, USA) was used for the rheological analysis of the oleogels. Specifically, a parallel plate geometry with a diameter of 40 mm was employed, and a gap of 700 μm was selected based on initial trials using gap distances from 1000 to 500 μm. The linear viscoelastic region (LVR) was detected through amplitude sweep (0.001–100%) at 1 Hz and 25 °C [57]. Additionally, frequency sweep experiments (0.1–10 Hz or 6.283 to 62.83 rad/s) were subsequently conducted at 25 °C, with the strain controlled within the LVR. The frequency dependency of G′ was analyzed by the *Power Law* model as follows:

(2) $$G^{’}=n\times \omega^{m}$$

where *n* is the constant coefficient (dimensionless); *ω* is the frequency (Hz); and *m* is the slop of frequency dependence of G′ or the flow behavior index (dimensionless) [58,59]. The viscoelastic parameters were also determined using temperature ramp experiments, which were performed in the range of 20–80 °C with a heating rate of 2 °C/min [57,60].

#### 4.4.4. Penetration Experiments

A Universal Testing Machine (Instron 3400, Norwood, MA, USA), equipped with a 50 N load cell, was employed for the determination of textural attributes of the oleogels. Glass tubes (28 mm diameter, 85 mm height) were filled with 15 mL of freshly prepared oleogels and stored at 4 °C. The penetration test was performed by lowering a cylindrical probe (11 mm diameter) at a penetration distance of 20 mm and a penetration speed of 100 mm/min [56]. The maximum force was recorded and expressed as firmness (N).

#### 4.4.5. Statistical Analysis

All results are shown as average ± standard deviation. Analysis of variance (ANOVA) was performed to determine the statistical differences with Excel software (Microsoft^®^ Excel^®^ 2019 version 2408). Tukey’s HSD (honest significant difference) test was used to indicate significant differences at a significance level of 5% (*p* < 0.05).

## Figures and Tables

**Table 1:** Kinetic parameters of the wax esters produced by spent coffee grounds oil with behenyl or cetyl alcohols, under solvent-free and solvent (limonene) conditions. Different superscript letters across the column indicate significant differences (*p* < 0.05).

Reaction Conditions	Wax Esters	Rate Constant (%/h)	R^2^
Solvent	Cetyl	13.88 ± 0.56 ^a^	0.96 ± 0.00
	Behenyl	14.33 ± 0.86 ^a^	0.94 ± 0.02
Solvent-free	Cetyl	22.41 ± 1.79 ^b^	0.98 ± 0.02
	Behenyl	23.55 ± 1.18 ^b^	1.00 ± 0.00

**Table 2:** Color parameters for olive oil oleogels structured with varying concentrations of behenyl wax esters produced under solvent-free conditions (B) and behenyl wax esters produced under limonene-solvent conditions (BL). Different superscript letters across each column indicate significant differences (*p* < 0.05).

Oleogels (% Wax Ester)	L*	a*	b*	C*	h°
B5	52.71 ± 1.78 ^a^	−1.89 ± 0.22 ^a^	20.41 ± 1.84 ^a^	20.49 ± 1.84 ^a^	95.31 ± 0.60 ^a^
B7	53.91 ± 0.21 ^a^	−2.54 ± 0.11 ^a^	18.74 ± 0.07 ^a^	18.92 ± 0.09 ^a^	97.73 ± 0.29 ^a^
B10	55.75 ± 0.37 ^a^	−2.25 ± 0.15 ^a^	18.12 ± 0.18 ^a^	18.26 ± 0.16 ^a^	97.10 ± 0.53 ^a^
BL5	57.09 ± 1.96 ^a^	−1.88 ± 0.75 ^a^	18.52 ± 5.31 ^a^	18.61 ± 5.26 ^a^	95.78 ± 2.52 ^a^
BL7	53.65 ± 2.54 ^a^	−2.20 ± 0.07 ^a^	19.09 ± 0.74 ^a^	19.22 ± 0.73 ^a^	96.59 ± 0.45 ^a^
BL10	56.07 ± 2.95 ^a^	−2.08 ± 0.28 ^a^	20.39 ± 0.02 ^a^	20.49 ± 0.02 ^a^	96.11 ± 0.51 ^a^

**Table 3:** Viscoelastic behavior during strain sweep tests and *Power Law* model parameters during frequency sweep tests for olive oil oleogels structured with varying concentrations of behenyl wax esters produced under solvent-free conditions (B) and behenyl wax esters produced under limonene-solvent conditions (BL). Different superscript letters across each column indicate significant differences (*p* < 0.05).

Oleogels (% Wax Ester)	Strain Sweep Parameters	*Power Law* Model Parameters		
	G′_LVR_ (kPa)	*n* (kPa∙s)	*m*	R^2^
B5	25.20 ± 5.85 ^a^	11.31 ± 3.76 ^a,b^	0.07 ± 0.01 ^a^	0.96 ± 0.00
B7	68.53 ± 6.25 ^b^	7.23 ± 0.22 ^a^	0.08 ± 0.02 ^a^	0.93 ± 0.03
B10	184.36 ± 8.72 ^c^	249.52 ± 18.33 ^c^	0.07 ± 0.00 ^a^	0.96 ± 0.01
BL5	22.43 ± 4.30 ^a^	21.61 ± 2.06 ^b^	0.05 ± 0.00 ^a^	0.92 ± 0.01
BL7	62.27 ± 17.72 ^b^	72.73 ± 13.52 ^b^	0.06 ± 0.00 ^a^	0.93 ± 0.02
BL10	256.24 ± 5.56 ^d^	278.80 ± 26.74 ^c^	0.05 ± 0.00 ^a^	0.94 ± 0.01

**Table 4:** Firmness of olive oil oleogels developed varying concentrations of behenyl wax esters (B, solvent conditions) and behenyl wax esters/limonene (BL, solvent-free conditions). Different superscript letters across the column indicate significant differences (*p* < 0.05).

Oleogel (% Wax Ester)	Firmness (Ν)
Β5	0.401 ± 0.075 ^a^
Β7	0.505 ± 0.007 ^a,c^
Β10	0.949 ± 0.093 ^b^
ΒL5	0.350 ± 0.032 ^a^
ΒL7	0.641 ± 0.038 ^c^
ΒL10	0.881 ± 0.039 ^b^

## Data Availability

The original contributions presented in the study are included in the article, further inquiries can be directed to the corresponding author.
